# Immunogenicity of an Inactivated Canine Adenovirus Type 1 Vaccine for Foxes

**DOI:** 10.3389/fvets.2022.678671

**Published:** 2022-02-15

**Authors:** Yang Fu, Jie Sun, Shizhen Lian, Xiaoyu Deng, Lei Zhang, Jikai Shao, Hongguang Yu, Xijun Yan, Yanzhu Zhu

**Affiliations:** ^1^Institute of Special Animal and Plant Sciences, Chinese Academy of Agricultural Sciences, Changchun, China; ^2^Department of Food Science and Chemical Engineering, Veterinary Medicine, Heze Vocational College Heze, Heze, China; ^3^Yuncheng No. 1 Middle School of Shandong Province, Heze, China; ^4^The Second Experimental Ocean School of Jilin Province, Jilin, China

**Keywords:** canine adenovirus type 1, silver fox, inactivated vaccine, immunogenicity, CAdV-1 F1301 strain

## Abstract

Canine adenovirus type 1 (CAdV-1) is the etiologic agent of fox encephalitis. As with most viral agents, the best method of prevention is vaccination. In this study, the CAdV-1 strain F1301 strain was used to construct a new type 1 canine adenovirus inactivated vaccine candidate, and its safety and immunogenicity were evaluated in silver foxes. Next, animals were challenged and survival rates of animals vaccinated with either the commercially available or the current candidate vaccine were examined. The results confirmed that the inactivated CAdV-1 vaccine prepared in this study can effectively protect against challenge with virulent CAdV-1 in silver foxes, and the safety profile was improved relative to that of the commercial vaccine. This study confirmed that the fox CAdV-1 F1301 strain can be used as a platform for an inactivated CAdV-1 vaccine.

## Introduction

Canine adenoviruses belong to the mastadenovirus genus of mammalian adenoviruses and the Adenoviridae family, which are divided into canine adenovirus type 1 (CAdV-1) and canine adenovirus type 2 (CAdV-2). Genomic sequence homology between CAdV-1 and CAdV-2 is about 70% (1). Although structurally similar, CAdV-1 and CAdV-2 infect different tissues. CAdV-1 targets the digestive tract and causes infectious hepatitis, nephritis, encephalitis, and conjunctivitis (2). Conversely, CAdV-2 infects respiratory tissues and induces infectious laryngotracheitis (3). CAdV-1 infects a wide range of animals, including dogs, foxes, wolves (4), mountain dogs, bears, skunks, and guinea pigs. The virus consists of a non-enveloped icosahedral virion of 80 nm in diameter that replicates in the nucleus of the host cell. The CAdV-1 genome is double-stranded DNA approximately 31 kb in size. The morphological and structural characteristics of CAdV-1 are typical of adenoviruses. The protein capsid is composed of 252 subunits, including the hexons, pentons, and fibrils. The hexons are the major component of the viral capsid, which also provide the complement activating epitopes. from the pentons extend a filament capped with a ball structure, the surface of which is coated with hemagglutinin molecules. When the virus infects the host cell, the ball functions as the receptor binding protein to initiate fusion and entry.

CAdV-1 is the etiologic agent of fox encephalitis. Typical symptoms of CAdV-1 infection in acutely ill foxes are loss of appetite, overexcitability, muscle cramps, fever, vomiting, and diarrhea. In recent years, CAdV-1 still has a high prevalence in different species of foxes (5–7). The virus has been identified in Italian red foxes (8), British red foxes, Korean fennec foxes (9), and Norwegian arctic foxes (10). Although CAdV-1 has a worldwide distribution, species susceptibility to the virus varies, with silver foxes (Vulpes vulpes) being more susceptible than arctic foxes, and arctic foxes more susceptible than dogs (11).

As post-exposure therapeutic interventions are often times not highly efficacious or economically viable options, the most effective way to prevent this virus infection in foxes is through vaccination. At present, there are many vaccine products for canine adenovirus protection, but most of them are CAdV-2 based vaccines (12). However, the latest research shows that domestic dogs vaccinated with the CAdV-2 live attenuated vaccine will shed virus, which can cause result in transmission between foxes and dogs, even in vaccinated animals (13). An early live attenuated CAdV-1 vaccine prepared by Zhou et al. was demonstrated to be safe, highly immunogenic, and provided long-lasting immunity (14). However, the attenuated CAdV-1 vaccine can cause damage to the eye membranes and kidneys of vaccinated animals. Therefore, further exploration of CAdV-1 vaccines is crucial.

Here, the use of a CAdV-1 F1301 strain isolated from a naturally infected Chinese sliver fox and passaged in MDCK cells will be characterized as a potential vaccine candidate (15). This virus was inactivated by 0.2% of formaldehyde. Aluminum hydroxide was used as an adjuvant, as it has a track record of being safe, effective (16, 17), and induces strong immune responses (18).

## Materials and Methods

### Ethics Statement

The experimental procedures outlined below were reviewed, approved, and conducted in compliance with the guidelines of the IACUC of the hosting institution. The approved research protocol number is NO.ISAPSAEC-2021-35.

### Virus Preparation

Dulbecco's modified eagle's medium (DMEM) (Gibco, USA), supplemented with 100 U/mL penicillin, and 100 μg/mL streptomycin (Gibco USA) was used to cultivate the Madin-darby canine kidney cell line (MDCK) (ATCC, China) at 37°C in a 5% CO_2_ incubator (Thermo scientific, USA).

A CAdV-1 F1301 strain, isolated from a naturally infected sliver fox, was cultured in MDCK cells and used as a vaccine candidate strain. Briefly, confluent MDCK monolayers were washed with PBS and inoculated with CAdV-1 F1301 suspended in DMEM (Invitrogen, Carlsbad, CA, USA). After 36 h at 37°C under 5% CO_2_, the culture medium was changed to remove non-infectious viruses. The infected cells were maintained at 37°C and 5% (v/v) CO_2._ Cytopathic effects (CPEs) were evaluated daily by light microscopy (Nikon, Japan). When the CPE occurred in more than 90% of cells, the flask was subjected to 3 freeze/thaw cycles between−20 to 4°C. The cells were then collected and centrifuged at 4°C, 10, 000 × g for 15 min to collect the clarified virus-containing fluid. The titers were determined using the method of Reed and Muench and expressed as TCID_50_ units/mL.

### Vaccine Preparation

The titers of CAdV-1 F1301 propagated in MDCK cells were determined to be >10^7^ TCID_50_/mL was inactivated with 0.2% (v/v) formaldehyde at 37°C for 24 h. The vaccine containing aluminum hydroxide gel (Changchun Huayi Biological Technology Co., Ltd.) was prepared by adding the adjuvant to a suspension of inactivated virus mixed in a volume ratio of 5:2. The pH value was adjusted to 7.2–7.4, and stored at 4°C. The commercial CAdV-2 live attenuated vaccine was purchased from Jilin Teyan Biotechnology Co., Ltd.

### Physical Properties and Sterility Testing of Inactivated CAdV-1 Vaccines

The inactivated CAdV-1 vaccines samples were inoculated on thioglycolate medium (TG) and 0.5% broth medium, and the two media were cultured at 37 and 25°C, respectively. Bacterial growth was determined after 3–5 days incubation (19).

### Verification of Virus Inactivation

Aliquots of inactivated virus solution were batch tested to validated inactivation. Vaccine virus preparations were used to inoculate MDCK cells; the control group receives the same dose of CAdV-1 live virus. An indirect immunofluorescence test (IFA) was used to detect infectious virus.

### Determination of Formaldehyde Content in Inactivated Virus Solution

A formaldehyde concentration standard of 0.1 mg/mL was used to compare experimental data. The inactivated virus solution was diluted 10 times with ultrapure water as the sample to be tested. From each standard and test sample, 0.5 mL was removed and added to the test buffer (10.0 mL of acetic acid-ammonium acetate buffer, 10.0 mL of acetone solution), and incubated at 60°C for 15 min, then rinsed with cold water for 5 min, and let stand at room temperature for 20 min. Absorbance at a wavelength of 410 nm was measured and calculated by the automated microtiter plate reader (Bio-Rad, USA). Formaldehyde solution (40%) content % (g/mL) = 0.25 × (absorbance of the sample solution to be tested/absorbance of the standard solution) %.

### Safety Assessment of Inactivated CAdV-1 Vaccine

The inactivated CAdV-1 vaccine (inactivated CAdV-1 + aluminum gel saline, 5:2, 3 mL) and PBS (PBS + aluminum gel saline, 5:2, 3 mL) was twice injected into the hind limbs of healthy silver foxes in the immunized group and control group (*n* = 6) with a 2-week interval. The physical condition, body temperature, and food intake of the animals were observed daily, for 10 days following immunization.

### Evaluation of Inactivated CAdV-1 Vaccine Immunogenicity in Fox

#### Animal Experiments

Healthy silver foxes aged 2–3 months, provided by the Zuojia Experimental Animal Base of the Specialty Research Institute, all animals were negative for CAdV-1, CDV, and CPV antigens and antibodies by PCR and serum neutralization tests, were used for vaccination and challenge studies. Healthy silver foxes (*n* = 24, Negative to CAdV-1 and CAdV-2) at the age of 2–3 months were selected and randomly divided into 3 groups, the inactivated CAdV-1 vaccine group, the commercial CAdV-2 attenuated live vaccine group, and control group, with 8 animals in each group. The commercial CAdV-2 attenuated live vaccine (in 1 mL) and inactivated CAdV-1 F1301 with aluminum hydroxide-adjuvant vaccines (5:2, in 1 mL) were intramuscularly injected into silver foxes. The foxes of the control group were injected with the same volume of PBS with aluminum gel saline. Vaccines (5:2, in 1 mL) were given intramuscularly, twice, with a 2-week interval. On days 0, 7, 14, 21, and 30 days post vaccination, blood was collected from all foxes. From 0 to 7 days post-vaccination, rectal swabs were collected from all animals, mixed with 0.5 mL PBS, and stored at−20°C. On the 30th day post-vaccination, all foxes were challenged with the CAdV-1 F1301 strain. An inoculum containing 100 LD_50_ (10^5^ TCID_50_) of CAdV-1 was injected intramuscularly into the hind limbs of all animals. Within 0–7 days post challenge, rectal swabs were collected from all foxes daily, mixed with 0.5 mL PBS and stored at -20°C.

##### Detection of CAdV-1 Serum Neutralizing Antibodies

The “fixed virus-diluted serum” method was used to detect the CAdV-1 neutralizing antibody titers in silver fox serum. The original serum of the three group were added in the first row of the plate. 100 μl of original serum was diluted with double dilutions starting from 1: 2 with the row. 50 μl of 100 TCID_50_ was added into each well of the plate. The plate was incubated at 37°C for 1 h. The solution in the plate was transferred to another cell culture plate with confluent monolayer MDCK cells. 150 μl of maintenance media was added into each well of the plate. The cell plate was cultured for 96 h. The cytopthic effect in each well was observed and the Reed-Muench method was used to calculate serum titer.

### Detection of Virus Shedding Post-vaccination

The Easy pure viral DNA Kit (Beijing Quanshi Jin Company) was used to extract CAdV-1 DNA from silver fox rectal swabs, and qPCR was used to detect CAdV DNA. Primers used to detect the CAdV DNA are presented in Table 1. The thermocycling profile for the reaction consisted of an initial denaturation/enzyme activation at 95°C for 10 min was followed by 45 cycles of denaturation at 95°C for 15 s, annealing at 60°C for 1 min, and extension at 72°C for 1 min, with a final extension at 72°C for 5 min. Any CT value after 40 cycles was considered to be non-specific, and therefore negative.

**Table 1:** Primers used to detect the CAdV DNA content.

**Primer (probe)**	**Sequence (5' to 3')**
CAdV-F	5'-AGTAATGGAAACCTAGGGG-3'
CAdV-R	5'-TCTGTGTTTCTGTCTTGC-3'
CAdV-1	5'-FAM-TCAATCGTCTCAACTAAATGCCGTG-BHQ1-3'
CAdV-2	5'-TxR-TCAGTCATCTCAGCTCAATGCCGTG-BHQ1-3'

### Changes in Body Temperature and Survival Rates Following Vaccination and Lethal Challenge

After the challenge experiment, the rectal temperature of all animals was monitored at 9 am and 5 pm for 10 days. Group survival rates were calculated everyday at 10 days post challenge.

### Statistics

All experimental data were analyzed using SPSS 20.0 software. The body temperature, serum neutralizing antibody titers, and qPCR data were analyzed by One-way ANOVA, and the results were expressed as Mean ± SE. ^*^ indicates significant difference (*P* < 0.05), ^**^ indicates extremely significant difference (*P*< *0.01*).

## Results

### The Quality Test of the Fox Inactivated CAdV-1 Vaccine

**Figure 1:**
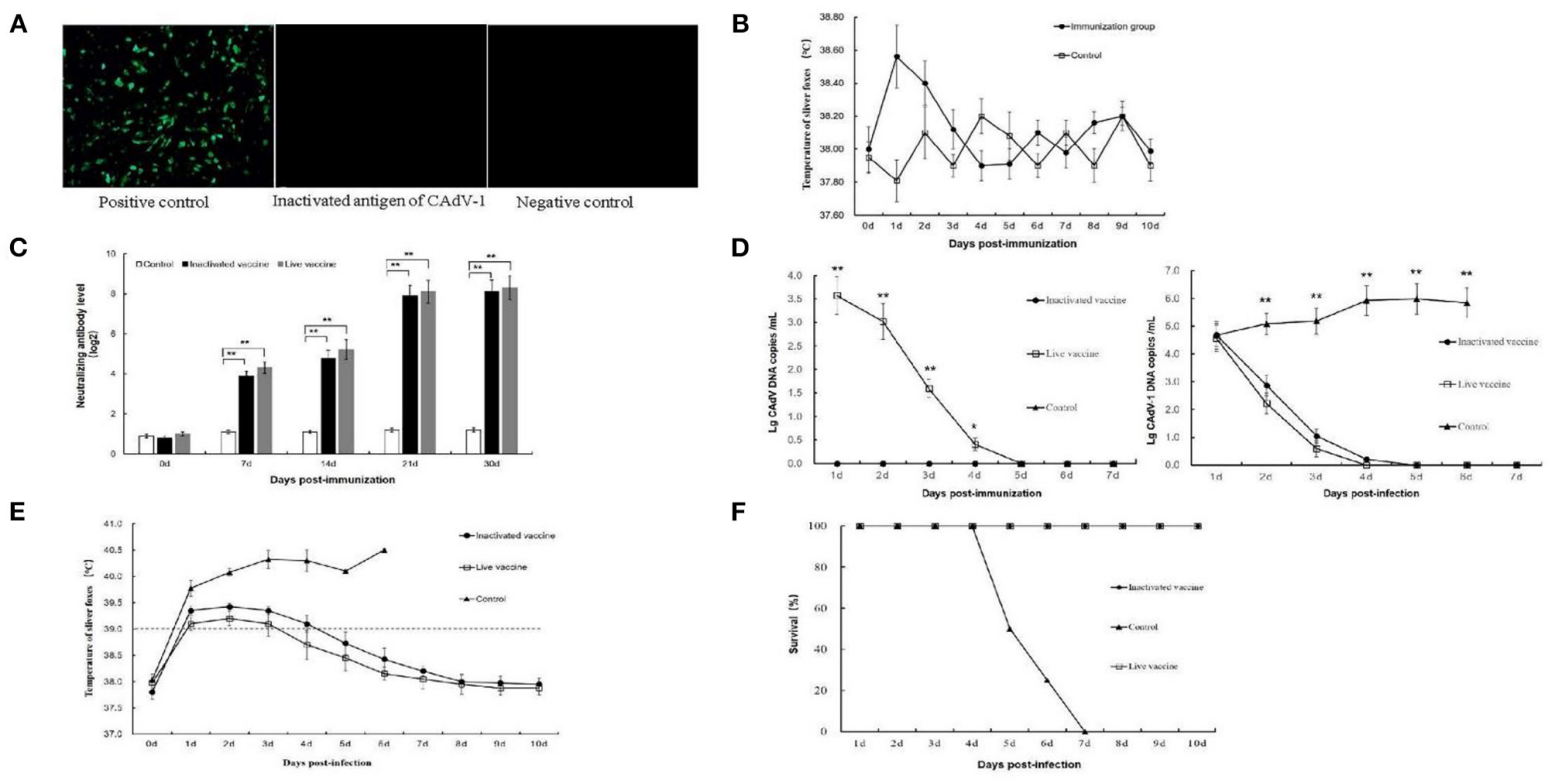
Immunogenicity of an inactivated canine adenovirus type 1 vaccine for foxes. **(A)** The indirect immunofluorescence result of an inactivated CAdV-1 antigen; **(B)** Silver foxes body temperature changes after immunization; **(C)** Serum neutralizing antibody titers; **(D)** Virus shedding detected by qPCR; **(E)** Silver foxes body temperature changes post infection; **(F)** The protective efficacy of the inactivated vaccine and the attenuated live vaccine in the infection experiment. **Means significant difference.

The titer of CAdV-1 for vaccine construction was 10^7.8^TCID_50_. The inactivated CAdV-1 vaccine with aluminum hydroxide gel adjuvant was light red suspension after being stored for a month at 4°C, with a small amount of milky white precipitated. There was no bacterial growth in the two mediums, indicating that the inactivated CAdV-1 vaccine with aluminum hydroxide gel adjuvant was sterilized. The positive control group showed a lot of green fluorescence; the inactivated CAdV-1 group showed no fluorescence; the negative control group did not show fluorescence (Figure 1A). The formaldehyde content in the inactivated antigen prepared meeted the standard and could be applied to animal experiments.

### Safety of Inactivated CAdV-1 Vaccine in Animals

Clinical signs and food intake of silver foxes receiving either the inactivated CAdV-1 or live attenuated CAdV-2 vaccines appeared healthy and normal, and no apparent differences were observed between the two groups. Rectal body temperatures of animals receiving the inactivated CAdV-1 vaccine was higher than that in the control group on the 1st and 2nd day after vaccination, which then stabilized and returned to normal by day 3 (Figure 1B). The results indicate that the inactivated CAdV-1 vaccine was safe.

### Immunogenicity of Inactivated CAdV-1 Vaccine in Animals

At all time-points tested, the virus neutralizing antibody (VNA) titers of both groups of vaccinated animals were significantly higher than control group (*P*< *0.01*), although no significant difference was observed between the two groups (Figure 1C).

### Virus Shedding Following Vaccination and Lethal CAdV-1 Challenge

From 1 to 4 dpv, the viral DNA loads of CAdV-2 in rectal swabs of animals in the live vaccine group were higher than those of the inactivated CAdV-1 vaccine group (*P*< *0.05*). No viral DNA of CAdV-1 was detected in animals that received the inactivated CAdV-1 vaccine, indicating that the inactivated CAdV-1 vaccine virus was not shed (Figure 1D). From 1–3 days post infection (dpi), the virus shedding ocured and decreased with the time in both live CAdV-2 vaccine group and inactivated CAdV-1 vaccine group. Through 5 dpi, rectal swabs in the both groups were negative for CAdV-1 DNA.

### Changes in Body Temperature and Survival Rate Following Lethal CAdV-1 F1301 Challenge

On the first day, the body temperature of the foxes in the control group increased to above 39.5 °C and continued to rise until all animals succumbed to infection. The body temperature of the foxes in both vaccinated groups increased above 39°C and dropped below 39°C on the 4 and 5 dpi, respectively. On 6 dpi, the body temperature of both the live and inactivated vaccine groups returned to normal (Figure 1E).

In control group, all foxes succumbed by 7 dpi, and the mortality rate was 100%. In inactivated and commercial CAdV-1 vaccine groups, all animals survived until the end of the study. Both vaccines were successful at mitigating the effects of lethal virus challenge (Figure 1F).

## Discussion

In contrast to many viruses that are tightly host restricted, CAdV-1 infects a wide range of animals, include dogs and foxes. In recent years, CAdV-1 has been highly prevalent in different species of foxes worldwide (5–7), including Italian red foxes (8), British red foxes, Korean fennec foxes (9), and Norwegian arctic foxes (10). Unfortunately, CAdV-1 is not sensitive to most disinfectants, so prevention of transmission via fomites is exceptionally challenging. Therefore, the most effective way to prevent infection of foxes is vaccination. At present, there are many vaccine products for canine adenovirus protection, but most of them protect against CAdV-2. However, the latest research shows that domestic dogs vaccinated with the CAdV-2 attenuated live vaccine will shed virus. This may lead to cross species infection (which may be sub-clinical) from dogs to foxes that come into direct contact with each other. Furthermore, only CAdV-1, but not CAdV-2, was detected in dead animals (13). Therefore, the CAdV-2 attenuated live vaccine still has risks, and seems to lack cross-protective efficacy against CAdV-1 infection. The fox CAdV-1 F1301 strain isolated from a naturally infected Chinese silver fox is a highly stable strain and can be cultured to very high titers (10^8^TCID_50_/ml) in MDCK cells. CAdV-1 F1301 strain meets the standards of “Chinese Veterinary Medicine,” which makes it a desirable platform from which to construct inactivated CAdV-1 vaccines. According to the physical characteristics of the CAdV-1 virus, and referring to the state of “Chinese veterinary pharmacopeia,” a final concentration of 0.2% formaldehyde was used to completely inactivate the virus.

The virus strain of the inactivated vaccine must be completely inactivated and its immunogenicity must be preserved as much as possible. The selection of an optimal adjuvant is essential when seeking to enhance and direct the immune response to vaccination. The aluminum hydroxide gel is stable, inexpensive, and is already in wide use worldwide. In addition, aluminum hydroxide gel can adsorb antigens on its surface to enhance immunity. Therefore, aluminum hydroxide as the adjuvant is well suited to compensate for the typical poorly immunogenic nature of inactivated vaccines (20).

The inactivated CAdV-1 vaccine developed here was safe and efficacious in animals, complied with the sterile standard, and the residual formaldehyde meets the standards of “Chinese Veterinary Medicine.” The physical condition and appetite of the vaccinated animals were normal, and a mild transient spike in body temperature, which lasted 2 days, was observed. In addition, detection of CAdV DNA in rectal swabs of vaccinated animals revealed shedding of the commercial live-attenuated vaccine (1-4dpv). No virus DNA was detected in the inactivated CAdV-1 vaccine group (*P* < 0.01).

The neutralizing antibody titers of the inactivated CAdV-1 vaccinated animals reached a peak on the 30 dpv, with no statistical difference being observed between the live-attenuated and inactivated vaccine groups (*P* > 0.05). After inoculation with a lethal CAdV-1 challenge, all silver foxes were shedding virus within 1–3 days and did not until the fifth day. The body temperature of silver foxes initially increased, decreased to normal by 6 dpv. The above experimental results demonstrate the safety and efficacy of the inactivated CAdV-1 vaccine, and it was similar to the commercial vaccine, both of which exhibited protective efficacy in silver foxes.

In summary, we created a new inactivated CAdV-1 vaccine based on the F1301 strain that was isolated from a naturally infected Chinese sliver fox. The inactivated CAdV-1 vaccine with the aluminum hydroxide adjuvant was safer in silver foxes than the commercially available live vaccines. Therefore, the fox-derived CAdV-1 F1301 strain appears to be a promising vaccine platform candidate to protect against natural infection in both farms and the wild.

## Data Availability Statement

The raw data supporting the conclusions of this article will be made available by the authors, without undue reservation.

## Ethics Statement

The animal study was reviewed and approved by IACUC of the hosting institution.

## Author Contributions

YF and JSu contributed equally to conduct and write this manuscript. YF, JSu, and SL conduct the construction of CAdV-1 inactivated vaccine. YF, JSu, XD, and LZ conducted the evaluation of safety and immunogenicity of CAdV-1 inactivated vaccine in silver fox. JSh, XD, and LZ help to assist the experiment and correct the manuscript. XY and YZ provide grant for this experiment and review this manuscript. All authors contributed to the article and approved the submitted version.

## Funding

This work was supported by grants from the Science and Technology Department of Jilin province (20200402045NC).

## Conflict of Interest

The authors declare that the research was conducted in the absence of any commercial or financial relationships that could be construed as a potential conflict of interest.

## Publisher's Note

All claims expressed in this article are solely those of the authors and do not necessarily represent those of their affiliated organizations, or those of the publisher, the editors and the reviewers. Any product that may be evaluated in this article, or claim that may be made by its manufacturer, is not guaranteed or endorsed by the publisher.
